# Finding of situs inversus in endoscopic sleeve gastroplasty: Case report

**DOI:** 10.1016/j.ijscr.2024.110727

**Published:** 2024-12-09

**Authors:** C.D. Quiroz Guadarrama, F. Girón, M. Rojano, G. Lopez-Nava, N. Zundel

**Affiliations:** a Clínica del Noroeste, Hermosillo, Mexico; b Faculty of Medicine, Universidad de los Andes, Bogotá, Colombia; c IFSO, USA; d Fundación Santa Fe de Bogotá, Colombia; e Hospital Sanchinarro, Madrid, Spain

**Keywords:** Situs Inversus, Obesity, ESG, Bariatric surgery, Case report

## Abstract

**Introduction and importance:**

Situs inversus is an anatomical rare condition in which visceral organs are not located in its normal position, with a reversal anatomical orientation.

**Case presentation:**

We present a case of an 27-year-old male with a Body Mass Index (BMI) of 36.02 Kg/m2, who was programed for a Endoscopic Sleeve Gastroplasty (ESG), in which Situs inversus was documented.

**Clinical discussion:**

Situs inversus is an uncommon anatomical condition that complicates identifying surgical landmarks and performing procedures like ESG. Preoperative imaging, such as abdominal ultrasound or computed tomography, is critical for confirming the diagnosis and planning the intervention. In this case, ESG was successfully executed using standard techniques, emphasizing the importance of expertise and careful planning. The reversed anatomy required adjustments in endoscopic navigation but did not necessitate major deviations from established protocols. This case highlights that, under the care of experienced endoscopists, ESG remains a safe and effective option for patients with situs inversus.

**Conclusion:**

Situs inversus is rare anatomical variation that can represent a challenge in bariatric endoscopic procedures such as ESG. Nevertheless, ESG can be safely performed under an experienced endoscopic bariatric surgeon.

## Highlights

• Situs inversus it’s a rare anatomical variation, reaching just 0,01% of the cases.
• Situs Inversus should be in the knowledge of bariatric surgeon
• Situs Inversus poses a surgical challenge; a skilled, multidisciplinary bariatric team is essential for achieving optimal outcomes.

## 1 Introduction

*Situs inversus* is a congenital condition in which the internal organs are arranged in a mirror-image orientation compared to the standard anatomical layout [[1], [2], [3]]. This condition can affect one or multiple organs, and its underlying genetic causes remain largely unclear. While sporadic mutations are thought to account for most cases, several inheritance patterns, including autosomal recessive, autosomal dominant, and X-linked recessive, have also been proposed [4,5]. The prevalence of *situs inversus* is estimated to occur in approximately 1 in 10,000 to 1 in 20,000 individuals [6].

The World Health Organization has reported that over 500 million adults are classified as obese, with nearly 2 billion categorized as overweight, underscoring the growing global obesity crisis [7]. Obesity is strongly associated with various preventable health conditions, including type 2 diabetes, hypertension, and cardiovascular disease. Bariatric surgery has become a cornerstone in managing these conditions. Recently, endoscopic bariatric procedures have gained popularity as a less invasive option for patients seeking alternatives to traditional surgery. Among these, Endoscopic Sleeve Gastroplasty (ESG) is widely recognized for effectively treating morbid obesity, promoting substantial weight loss, and improving overall health outcomes.

We present a case of a 27-year-old male with obesity with a BMI of 36.02 Kg/m2, no previous medical history, who underwent Endoscopic Sleeve Gastroplasty (ESG). Work has been reported in accordance to SCARE guidelines [8].

## 2 Case presentation

After ethical and institutional approval, previous informed consent filled, following SCARE guidelines [3]. A 27-year-old male presented with a history of progressive weight gain, despite several dietary modifications and exercise programs, which initially led to weight loss but was followed by weight regain, reaching a maximum weight of 118 kg. His initial physical examination was unremarkable. A comprehensive preoperative workup, including assessments by psychiatry, internal medicine, and nutrition, revealed no abnormalities.

**Fig. 1:**
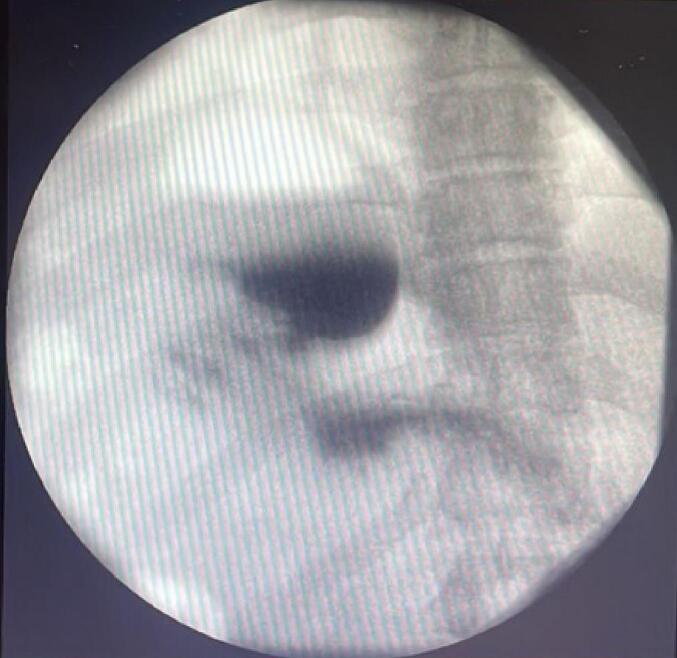
Right gastric fundus in radiologic evaluation.

The patient was subsequently referred to our bariatric surgery department, where his case was discussed in a multidisciplinary meeting. After thorough evaluation, the team recommended Endoscopic Sleeve Gastroplasty (ESG). Under general anesthesia, an initial diagnostic endoscopy was conducted at the start of the procedure to rule out any potential lesions. The right fundus was visualized during the examination, with no other abnormalities detected (Fig. 1). An esophageal over tube was utilized to facilitate the smooth passage of the suturing device and to help retain gas. Endoscopic suturing system was used for suturing, employing a 2-0 polypropylene thread.

**Fig. 2:**
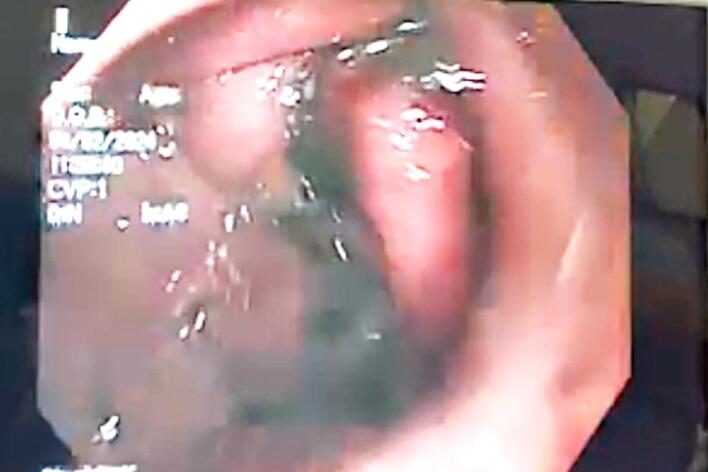
Endoscopic view. *Greater curvature. **Lesser curvature.

A U-shaped rectangular suture pattern was applied in the following manner: starting on the anterior wall, 4–6 sutures were placed along the greater curvature extending toward the posterior wall, and then 4–6 sutures were placed from the posterior wall back toward the anterior. Five folds were created along the distal gastric body up to the fundus, ending approximately 2–3 cm below the gastroesophageal junction, resulting in gastric tubulization (Fig. 2). The procedure was completed without any complications.

Postoperatively, the patient experienced an uneventful recovery, requiring no additional interventions, and was discharged with standard postoperative care instructions, including adherence to a full bariatric diet. At the 6-month follow-up, the patient remained complication-free, achieving a Total Weight Loss (TWL) of 11.9 %, with a current BMI of 31.74 kg/m^2^. He has resumed normal daily activities and continues under the multidisciplinary care of the bariatric team.

## 3 Discussion

The global prevalence of obesity continues to rise, accompanied by an increase in obesity-related comorbidities such as diabetes, hypertension, and cardiovascular disease [7]. While traditional dietary interventions and lifestyle modifications remain foundational, they often prove insufficient for advanced stages of obesity. Bariatric surgery has emerged as a cornerstone in managing morbid obesity, offering effective and sustainable outcomes [9,10]. Among surgical options, gastric bypass and vertical sleeve gastrectomy are the most commonly performed procedures. However, endoscopic techniques, such as Endoscopic Sleeve Gastroplasty (ESG), have gained traction as minimally invasive alternatives for select patient populations [11].

ESG employs a transoral approach using a full-thickness endoscopic suturing system to create a gastric sleeve, effectively reducing stomach volume and delaying gastric emptying. In the MERIT trial, Barham et al. [11] demonstrated the efficacy of ESG in achieving significant weight loss and improved metabolic control in patients with grade I and II obesity, positioning ESG as a versatile option for both bridging therapy and primary intervention. Its minimally invasive nature, reduced recovery time, and safety profile make ESG an appealing option in the evolving landscape of bariatric treatment [11].

Situs inversus totalis, characterized by the complete mirror-image reversal of internal organ positioning, presents unique challenges in surgical and endoscopic procedures. This condition, often asymptomatic, may go undetected until adulthood unless accompanied by congenital anomalies or cardiac defects [[1], [2], [3]]. It is typically discovered incidentally through imaging studies or electrocardiograms performed for unrelated reasons. Given that patients may be unaware of their condition, surgeons must conduct thorough medical histories and inquire specifically about congenital anomalies or atypical organ positioning [12,13]. A detailed physical examination, including auscultation for dextrocardia, can raise suspicion, while imaging modalities like ultrasound or chest X-ray can confirm the diagnosis [[12], [13], [14]].

Situs inversus can manifest in varying degrees, from complete transposition to partial forms affecting specific organs. Partial situs inversus necessitates precise identification of the involved organs to inform surgical planning. Moreover, this condition is frequently associated with congenital anomalies, particularly cardiac defects such as atrial or ventricular septal defects and abnormalities in the great vessels or valves, underscoring the need for preoperative echocardiography and cardiovascular evaluations [[1], [2], [3], [4]].

In the context of bariatric surgery, situs inversus poses unique anatomical and technical challenges. The mirrored anatomy complicates the identification of anatomical landmarks and may require adaptations during procedures like suturing. While ESG has been deemed safe for patients with situs inversus [15], it demands meticulous preoperative planning and surgical expertise. Neto et al. [15] reported that while minor adjustments were needed, no significant deviations from standard ESG techniques were required, a finding that aligns with our experience. In our case, ESG was successfully performed without significant alterations to the procedure, highlighting its feasibility in this population.

Comparing our case with others reported in the literature reveals common challenges in managing situs inversus during bariatric procedures. For instance, cases by Almussallam et al. [13] and Burvill et al. [14] described laparoscopic sleeve gastrectomy in patients with situs inversus totalis, emphasizing the importance of detailed preoperative imaging and intraoperative flexibility to navigate the mirrored anatomy. Although these studies focused on laparoscopic techniques, their principles of thorough preparation and adaptability are equally applicable to endoscopic interventions.

A distinctive aspect of our case is the application of ESG in a patient with situs inversus, contributing to the limited body of evidence on endoscopic bariatric procedures in this unique population. While laparoscopic procedures in situs inversus have been more extensively documented, data on ESG remain sparse. Our findings suggest that ESG can be performed successfully without compromising patient safety or outcomes, even in the presence of reversed organ anatomy.

Further research is essential to validate the efficacy and safety of ESG in situs inversus patients. Long-term studies focusing on weight loss, metabolic outcomes, and complication rates will provide valuable insights. Additionally, accumulating case reports will help refine procedural techniques and improve the understanding of the challenges associated with bariatric interventions in this rare condition.

By comparing our experience with previously reported cases, we underscore both the shared challenges and unique opportunities in managing situs inversus during bariatric procedures. This highlights the importance of individualized surgical planning and contributes to the growing body of knowledge on minimally invasive bariatric interventions in anatomically complex patients.

## 4 Conclusion

Situs inversus is a rare anatomical variation that can pose significant challenges in bariatric endoscopic procedures like endoscopic sleeve gastroplasty (ESG). However, with the expertise of an experienced endoscopic bariatric surgeon, ESG can be performed safely and effectively, ensuring optimal outcomes for patients despite this anatomical anomaly.

## Author contribution

**Felipe Giron, MD, MSc**: Make substantial contributions to conception and design, acquisition of data,analysis and interpretation of data.

**Martin Rojano, MD**: Participate in drafting the article and revising it critically for important intellectual content.

**Gontrad Lopez Nava, MD**: Participate in drafting the article and revising it critically for important intellectual content.

**Natan Zundel, MD**: Participate in drafting the article and revising it critically for important intellectual content.

**Cesar David Quiroz Guadarrama, MD**: Participate in drafting the article and revising critically for important intellectual content. Give final approval of the version to be submitted and any revised version.

## Consent

Written informed consent was obtained from the patient for publication of this case report and accompanying images. A copy of the written consent is available for review by the Editor-in-Chief of this journal on request.

## Ethical approval

Ethical approval of institutional committee was made previous publication.

## Research registration number

None.

## Provenance and peer review

Not commissioned, externally peer-reviewed.

## Funding

This research did not receive any specific grant from funding agencies in the public, commercial, or not-for-profit sectors.

## Conflict of interest statement

Authors do not declare any conflict of interest.
